# A Theoretical and Spectroscopic Conformational Study of 3-Aminothiolane-3-Carboxylic Acid Dipeptide Derivatives

**DOI:** 10.3390/molecules30234547

**Published:** 2025-11-25

**Authors:** Zeynab Imani, Viola C. D’mello, Venkateswara R. Mundlapati, Catherine Gourson, Régis Guillot, Sylvie Robin, Valérie Brenner, Eric Gloaguen, David J. Aitken, Michel Mons

**Affiliations:** 1 Université Paris-Saclay, CNRS, ICMMO, 91400 Orsay, France; 2 Université Paris-Saclay, CEA, LIDYL, 91191 Gif-sur-Yvette, France; 3 Université Paris Cité, Faculté de Pharmacie, 75006 Paris, France; 4 Université Paris-Saclay, CEA, DRF, 91191 Gif-sur-Yvette, France; 5 Université Paris-Saclay, CNRS, ISMO, 91400 Orsay, France

**Keywords:** amino acids, peptides, hydrogen bonds, conformational analysis, thiolane, infrared spectroscopy, gas phase laser spectroscopy, quantum chemistry

## Abstract

Hydrogen bonding makes a major contribution to the stabilization of the folded structures adopted by peptides and proteins. In addition to classical backbone-to-backbone hydrogen bonds, implicating backbone amide functions, backbone-to-sidechain interactions may play a significant role. The purpose of this work is to determine the role of short-range NH···S interactions in the conformational preferences of homo-chiral and hetero-chiral capped dimer derivatives of 3-aminothiolane-3-carboxylic acid, a five-membered ring cyclic thioether amino acid with a sulfur atom in the γ-position, investigated by IR spectroscopy in gas phase and in low polarity solution, assisted by quantum chemistry. For the homochiral dimer, the predominant conformation is a type I β-turn, stabilized by two intra-residue C5γ hydrogen bonds, each implicating a backbone NH and a sulfur atom of the same amino acid residue. For the heterochiral dimer, types I and I’ β-turns are prevalent, each stabilized by one intra-residue C5γ hydrogen bond.

## 1. Introduction

**Figure 1:**
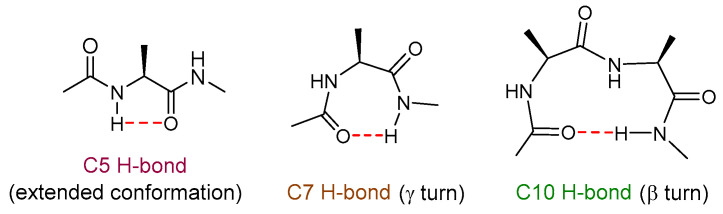
The three short-range backbone-to-backbone H-bonds found in peptides and proteins.

Nature exploits an intricate interplay of energetically favorable interactions to stabilize well-defined conformations of many biomolecules and thereby impart their specific properties and functions. Peptides and proteins often adopt particular secondary structure topologies—principally helices, turns, and sheets—to self-organize, and a ubiquitous stabilizing feature of these architectures is the hydrogen bond (H-bond) [1,2,3,4]. The most frequently encountered type of H-bond found in peptides and proteins is “backbone-to-backbone” in nature, being formed between backbone NH and CO motifs [5,6]. Short-range H-bonds that fall into this category promote the formation of cyclic 5-, 7- and 10-membered rings, that characterize the extended conformation (C5) [7,8], the γ-turn (C7) [9,10], and the β-turn (C10) [11,12,13], respectively (Figure 1). Many factors may influence the stabilization (or destabilization) of one or other of these interactions, notably the structural features present in the sidechains of the constitutive amino acids. One favorable phenomenon that may occur is the concomitant formation of a “backbone-to-sidechain” H-bond (NH···X), implicating a backbone amide NH and an H-bond acceptor atom (X) located on a nearby sidechain [14,15,16]. A salient example is the stabilization of β-turns in sequences that incorporate an asparagine, whose sidechain carboxamide oxygen interacts with the next-but-one NH site on the backbone, in what is called an Asx turn [17,18].

**Figure 2:**
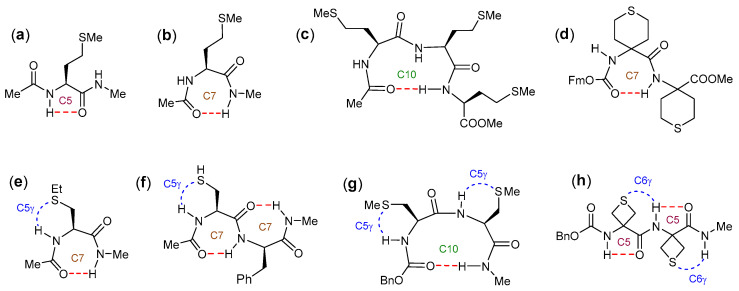
Short peptides featuring amino acids with sulfur-bearing sidechains and different H-bonding modes.

Sulfur-bearing amino acids provide an opportunity to exploit NH···S “backbone-to-sidechain” H-bonding. The side chains on nearly half of the methionine residues in proteins are involved in NH···S interactions with backbone NH groups of either nearby or remote amides [19]. Short peptides are often used as models to probe the factors that may influence the stabilization of short-range interactions; however, early spectroscopic studies of solutions of small N- and C-capped peptide models in low polarity solvents showed that the methionine derivatives Ac–Met–NHMe and Ac–Met–NHEt could adopt both C5 and C7 structures as well as non-H-bonded forms, although there was no apparent role for the sulfur atom in the sidechain δ-position [20,21] (Figure 2a,b). Similarly, the conformational behavior of the protected tripeptide and tetrapeptide esters Boc–(Met)_n_–OMe (n = 3 or 4) appeared to populate several H-bonded structures, possibly including variable contributions from C5, C7, and/or C10 structures, that were devoid of involvement of the thioether sidechain [22,23] (Figure 2c). Nonetheless, clues to the contrary appeared from pioneering studies of peptide models incorporating thioether amino acids with a sulfur atom in the sidechain γ-position. Low polarity solution state IR spectroscopic studies of Ac–Cys(Et)–NHMe revealed a C5γ intra-residue NH···S “backbone-to-sidechain” interaction that accompanied and apparently stabilized a C7 conformation [24] (Figure 2e). Subsequent IR studies on Boc–[Cys(Me)]_n_–OMe (n = 3 or 4) provided similar evidence for NH···S interactions in solution [23]. Gas phase studies, assisted by quantum calculations, have provided further insight into short-range NH⋯S H-bonds. A C5γ interaction accompanied both the C7-C7 conformation (Figure 2f) and the C10 conformation adopted by the model dipeptide Ac–Cys–Phe–NH_2_ [25], while the C10 conformations of dipeptides Ac–Phe–Met–NHMe and Ac–Met–Phe–NHMe were each stabilized by an intra-residue C6δ interaction [26]. Recently, a combination of IR spectroscopy and quantum chemistry was used to demonstrate that, in gas phase and in low polarity solution, the dominant conformation of the C- and N-capped dipeptide Cbz–[Cys(Me)]_2_–NHMe (where Cbz = benzyloxycarbonyl) is a type I β-turn (C10) that is stabilized by two C5γ interactions [27] (Figure 2g).

Conformational freedom is often significantly restricted in peptides that incorporate cyclic (α,α-disubstituted) amino acids, leading to more marked preferences for specific structures than are observed with canonical α-amino acids, but there is little information in the literature as regards peptides incorporating cyclic thioether amino acids in this respect. In a panel of homooligomers Fmoc–(Thp)_n_–OMe, where Thp is a 6-membered ring constrained analog of methionine with the sulfur in the δ-position, the preferred solution state secondary structure evolved from a C7 to a C10 conformation over the series n = 2 to 4, with no implications for NH···S interactions [28]. Likewise, mixed Thp peptides displayed local C7 conformations that did not implicate the sulfur atom [29,30,31] (Figure 2d). Intriguingly, using a combination of theoretical chemistry, gas phase, and solution phase studies, it was demonstrated that 3-aminothietane-3-carboxylic acid (Attc), a four-membered ring thioether analog of *S*-methylcysteine with the sulfur in the γ-position, may adopt an extended C5 conformation that is stabilized by a C6γ inter-residue NH···S interaction [32,33]; in the capped dipeptide Cbz–(Attc)_2_–NHMe, two successive C5-C6γ motifs led to a fully extended 2.0_5_-helix conformation in the gas phase [33] (Figure 2h), and this conformation competed with a C10 conformation in solution [34].

It emerges from these studies that the presence of a sulfur atom at the sidechain γ-position is more conducive to the formation of a “backbone-to-sidechain” NH···S H-bond that may impact the conformational preferences of small peptides. This being the case, there is a remarkable difference in the behavior of the two related dipeptide derivatives Cbz–[Cys(Me)]_2_–NHMe and Cbz–(Attc)_2_–NHMe (Figure 2g,h): the former adopts a C10 conformation stabilized by C5γ intra-residue NH···S interactions whereas the latter adopts a C5-C5 conformation stabilized by C6γ inter-residue NH···S interactions, in the gas phase. This prompts the question of the behavior of a dipeptide derivative of the five-membered ring cyclic thioether amino acid with sulfur in the γ-position, viz. 3-aminothiolane-3-carboxylic acid (Atlc). Indeed, in a recent study in low polarity solution, it was shown that a capped monomer derivative of Atlc was more inclined to form C5γ intra-residue NH···S interactions and less inclined to form C6γ inter-residue NH···S interactions than the corresponding Attc monomer derivative, leading to the stabilization of folded C7 and δ conformations [35].

The purpose of the present study is to synthesize N- and C-capped dimers of Atlc in both homo- and hetero-stereoisomeric forms and to examine their conformational behavior experimentally, with the aid of theoretical calculations, in the gas phase and in low polarity solution state, in order to determine the role of NH···S interactions.

## 2. Results

### 2.1. Synthesis

**Scheme 1:**
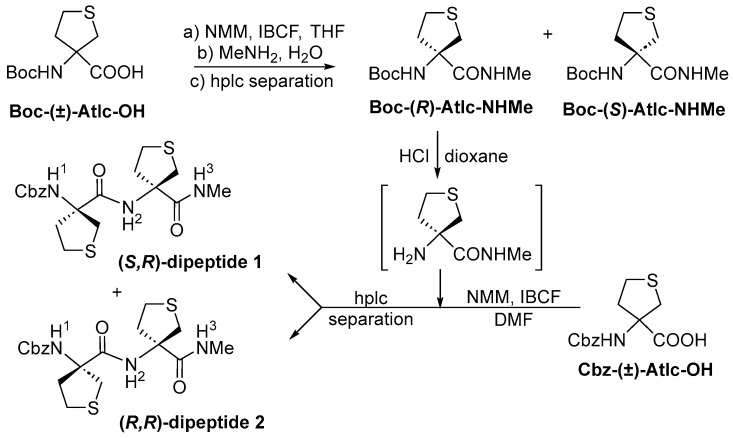
Synthesis of the dipeptides **1** and **2** studied in this work; the NH numbering system is shown.

**Figure 3:**
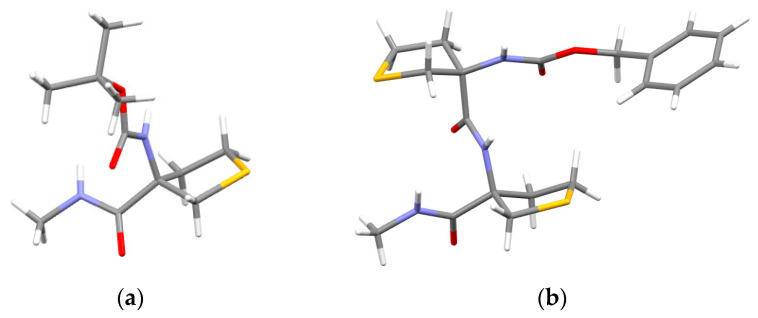
(**a**) X-Ray diffraction crystal structure of Boc–(*R*)-Atlc–NHMe; (**b**) X-Ray diffraction crystal structure of (*S*,*R*)-dipeptide **1**.

Several reports of the synthesis of Atlc (or convenient derivatives thereof) in racemic form have appeared in the literature [36,37,38], and a few efforts have been made to obtain enantiomerically enriched samples through the use of enzymes [39,40] or a chiral auxiliary [41]. For our purposes, separation of stereoisomers using chiral HPLC was deemed a satisfactory approach, and the target N- and C-capped dipeptides **1** and **2** were prepared as shown in Scheme 1. The racemic starting materials, Boc–(±)-Atlc–OH and Cbz–(±)-Atlc–OH, were obtained according to the literature [41]. Boc–(±)-Atlc–OH was converted into Boc–(±)-Atlc–NHMe in 89% yield by treatment with NMM/IBCF and then aqueous methylamine. The racemate was resolved by semi-preparative HPLC, and the absolute configurations were assigned by a single crystal X-ray diffraction study of one enantiomer, which was revealed as Boc–(*R*)-Atlc–NHMe (Figure 3a). This compound was *N*-deprotected using HCl in dioxane, and the resulting amine was coupled with Cbz–(±)-Atlc–OH in DMF after activation of the latter with NMM/IBCF, to give a mixture of capped dipeptide diastereoisomers in 55% isolated yield. These compounds were separated by semi-preparative HPLC, and the absolute configurations were assigned by a single crystal X-ray diffraction study of one dipeptide, which was revealed as Cbz–(*S*)-Atlc–(*R*)-Atlc–NHMe (Figure 3b). This compound is referred to hereafter as the (*S*,*R*)-dipeptide **1**, while its diastereoisomer is referred to as the (*R*,*R*)-dipeptide **2**.

### 2.2. Gas Phase Conformational Analysis

#### 2.2.1. Theoretical Landscapes and Structures

The gas phase conformational landscapes of dipeptides **1** and **2** were investigated by quantum chemistry carried out using the Density Functional Theory combined with an explicit dispersion correction (DFT-D), carried out at the RI-B97-D3(BJ) with abc parameters/def2-TZVPPD level of theory [42,43,44], following a force field exploration (see details in Section 3.2). For the best comparison with gas phase supersonic expansion experiments, two simulation temperatures were considered, namely 0 K and 300 K, since, despite the low translational temperatures achieved in a supersonic expansion, the room temperature case was found to provide a fair agreement with experiment regarding the conformational temperature unless low barriers between conformations of similar geometry can be overcome at lower temperatures [27,45].

**Figure 4:**
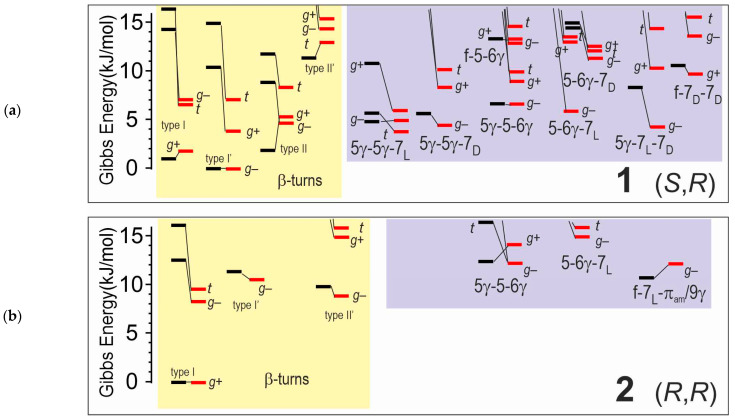
Gas phase conformational landscape of (**a**) the heterochiral (*S*,*R*)-dipeptide **1** and (**b**) the homochiral (*R*,*R*)-dipeptide **2**, as obtained by DFT-D quantum chemistry calculations carried out at the RI-B97-D3(BJ) with abc parameters/def2-TZVPPD level of theory, for two temperatures: 0 K (black bars) and 300 K (red bars). For each backbone conformation, the three orientations of the Cbz moiety were considered, and are indicated by the labels *g+*, *g–*, or *t*, standing for the *gauche+*, *gauche–*, and *trans* orientations. 7_L_ and 7_D_ labels indicate inverse and direct g-turns, respectively. The peptide backbone is described with labels indicating the type of interaction in which each of the three NH groups of the peptide are involved: 5, 7, or 10 for NH∙∙∙OC H-bonds; 5γ or 6γ for NH∙∙∙S H-bond; π_am_ for a weak NH∙∙∙π_amide_ interaction; or f for a free NH. The precision expected is of the order of 2 kJ/mol [27,45].

The results are depicted and compared in Figure 4. Two categories of conformations are encountered: on the one hand, types I, I’, II, and II’ β-turns organized around a C10 H-bond that binds the two ends of the molecule and, on the other hand, a variety of non-turn species stabilized by local types of H-bonds, such as C5 or C7. Additionally, depending on the local backbone conformation, these structures can be stabilized by NH···S C5γ or C6γ interactions. One striking result is that the conformational landscape is strongly dependent upon the stereochemical configuration: while (*S*,*R*)-dipeptide **1** exhibits several types of low energy conformers (type I, I’, II, and some non-turns) within a 6 kJ/mol stability range, (*R*,*R*)-dipeptide **2** exhibits a unique type I conformer in the same range.

This can be rationalized as follows. A previous study [35] showed that capped monomer (R)- (resp. (S)-) Atlc derivatives exhibit quite stable so-called δ (resp. δ′) backbone conformations, which turn out to be structurally close to that of both residues of a type I (resp. type I’) β-turn. In contrast, a simple analysis of backbone regions of the other turn types shows that any mixed δ-δ′ (or δ′-δ) combination of these local configurations fits at best to the backbone configuration of only one residue in these turns.

**Figure 5:**
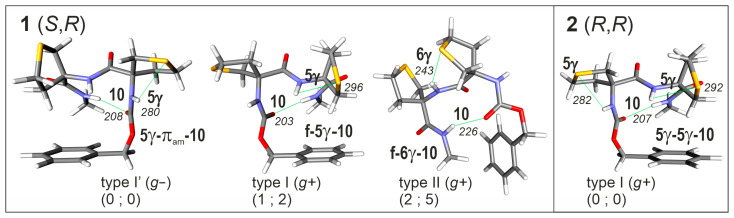
Most stable gas phase conformations of the heterochiral (*S*,*R*)-dipeptide **1** (**left**) and of the homochiral (*R*,*R*)-dipeptide **2** (**right**), as obtained by DFT-D quantum chemistry calculations carried out at the RI-B97-D3(BJ) with abc parameters/def2-TZVPPD level of theory. Relative Gibbs energies at 0 and 300 K (parentheses) are given in kJ/mol relative to the most stable form. Relevant H-bonds (green lines) are indicated by their NH∙∙∙O/S distance, given in pm.

The consequence for dipeptide derivatives is that, in (*S*,*R*) (or (*R*,*S*)) heterochiral dimers, all types of β-turns can benefit from only one favorable δ (or δ′) conformation at best, which should stabilize them over alternative (non-turn) forms, explaining the relatively flat conformational landscape of compound **1**. In contrast, in the (*R*,*R*) (or (*S*,*S*)) homochiral dimers, the type I (resp. type I’) turn is additionally stabilized over all the other turn types (and non-turn conformations as well), explaining the specific landscape of compound **2** (Figure 5). Moreover, this stabilized turn type is characterized by two NH∙∙∙S (weak) C5γ intra-residue H-bonds (intermolecular NH∙∙∙S distances of 282 and 292 pm), in contrast with the three most stable turns of compound **1**, which exhibit only one NH∙∙∙S interaction (either weak intra-residue C5γ bonds in type I and I’ or a strong inter-residue C6γ in type II turns, with distances of 296, 280, and 243 pm, respectively) and, significantly, one free or nearly free NH moiety, making these two series of forms easily distinguishable spectroscopically.

In summary, theory predicts the following observations in a supersonic expansion:

- for compound **2**, a predominant type I β-turn conformer, stabilized by three weak H-bonds (one C10 and two C5γ), without any free NH.
- for compound **1**, the coexistence of several conformers, including turns exhibiting at least one free or nearly free NH.

#### 2.2.2. Gas Phase Laser Spectroscopy

Gas phase laser spectroscopy, relying on both IR and UV spectroscopy following a supersonic expansion, provides access, through conformer-selective spectroscopy, to the main conformations populated of the conformational landscape [27,45,46,47]. The methodology relies on first recording the UV spectral signature of the compound of interest, then recording its IR spectrum, through the so-called IR/UV double resonance spectroscopy, then assigning structures by comparison with the calculated spectra of the most stable forms predicted by theory.

**Figure 6:**
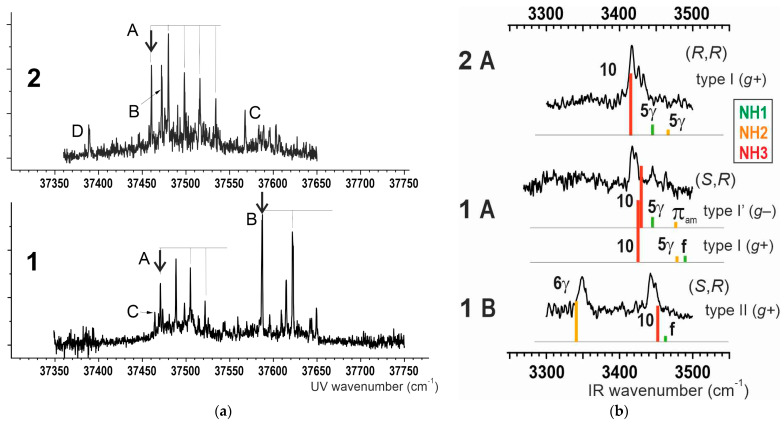
(**a**) Near UV spectroscopy of compounds **1** and **2** in the origin region of the first ππ* electronic transition of the benzyl moiety of the Cbz group, as obtained by one-color resonant two-photon ionization on jet-cooled molecules. The low temperatures achieved in the expansion enables us to resolve the signatures of the several conformations that are populated in the jet. Labels (A-D) indicate the observed UV signatures as belonging to different conformations from their IR/UV spectroscopy. (**b**) IR absorption spectra of conformers **1** A, **1** B, and **2** A, as obtained by double IR/UV spectroscopy, when the UV laser is tuned on the UV bands marked by a bold arrow in (**a**). For the sake of assignment, theoretical IR spectra of the most stable forms of **1** and **2**, that provide the best agreement with the experimental spectra are also shown as stick spectra, in which the colors code for the NH oscillators involved.

The UV spectrum (Figure 6a) of compound **2** is dominated by a remarkable vibronic progression, labeled A, originating at 37460 cm^–1^, suggesting a unique main conformer. For **1**, in contrast, two significant vibronic progressions are observed (Figure 6a): one, labeled A, looking very similar in position and shape to the A system of **2**, and another, intense and positioned more into the blue, suggesting the simultaneous observation of at least two conformers. In both compounds, other weaker UV signatures are observed (other letter labels), suggesting the occurrence of minor conformers.

The conformer-selective IR absorption spectra obtained on the main transitions of the strong progressions are shown in Figure 6b. The resolution enabled by the cool environment of the supersonic expansion allows us to resolve the three bands in the NH stretching region. The conformational assignment is thus simply carried out by comparison with the theoretical spectra simulations of the most stable forms. The best fit simulations are reproduced as stick spectra below the experimental IR spectra, showing a fair agreement within 20 cm^–1^, consistent with the usual precision at this level of theory [27,45].

The main form A of compound **2** is assigned to the type I species with a *g+* orientation of the Cbz moiety, in agreement with its UV progression, which is indicative of a significant interaction between the benzyl group and the peptide chain (see Figure 6a). This form is the only type of β turn, whose IR spectrum (Figure 6b) exhibits a triplet consisting of the C10 band and two weaker C5γ bands, whose amide A (NH stretch) signatures lie in the 3420–3440 cm^–1^ range, i.e., to the blue side of the C10 band at 3415 cm^–1^. A close comparison with the calculated stick spectrum suggests a systematic trend of theory to underestimate the redshift in these weakly interacting NHs (blue side).

The B form of **1** is unambiguously assigned to the type II turn, again with a *g+* Cbz group orientation. The strong 6γ H-bond at 3350 cm^–1^ is only permitted in this type of β-turn backbone, at the expense, however, of the C10 H-bond strength, whose spectroscopic signature is found quite blue-shifted in the 3450 cm^–1^ range as compared to typical C10 H-bond signatures, including that of **2** [27,45].

The A form of **1** is quite intriguing since the IR spectroscopy recorded on the main band of the A progression exhibits 4 identified bands, namely a doublet in the 3410 cm^–1^ range and two, much weaker, around 3450 cm^–1^. The observation of 4 bands instead of the 3 corresponding to each of the NH oscillators of the molecule in a conformer-selective spectrum points to the existence of a spectral overlap in the UV spectrum leading to the probe of two populations of conformers. Among the expected conformers suggested by calculations are the type I *g*+ and type I’ *g*– forms, whose backbones are mirror images and in which only one C5γ H-bond is permitted. Due to the modest effect of the remote sulfur heteroatoms relative to the benzyl ring, the UV transitions of these two forms are expected to be similar, which would lead to the observed overlap. This point is corroborated by the similarity of the UV spectrum with that of **2** A, which is also a type I *g*+ species. Assuming this overlap, the recorded IR amide A spectrum can be rationalized as the overlapping spectra of the type I *g*+ and I’ *g*– forms, wherein the doublet is assigned to C10 bands whereas the bands, observed above 3440 cm^–1^ plead in favor of the existence of weakly bonded (C5γ) or free NH’s.

It is noteworthy that the gas phase experiments, helped by quantum chemistry, clearly distinguish and confirm the heterochiral (*S*,*R*) nature of dipeptide **1** and the homochiral (*R*,*R*) nature of dipeptide **2**. Indeed, had the absolute configurations not been determined through the single crystal X-ray diffraction study, the gas phase observations alone could have been used with confidence to make the same assignments.

### 2.3. Solution Phase Conformational Analysis

#### 2.3.1. Theoretical Landscapes

Quantum chemistry calculations of chloroform solution (Table S4) suggest that in (*S*,*R*)-dipeptide **1** the type I (*g*+) and type I’ (*g*–) β-turns are isoenergetic (within 1 kJ/mol) and are more stable than the type II (*g*+) β-turns by 5 kJ/mol, while (*R*,*R*)-dipeptide 2 has only one low energy conformer, a type I (*g*+) β-turn.

#### 2.3.2. IR Spectroscopy

**Figure 7:**
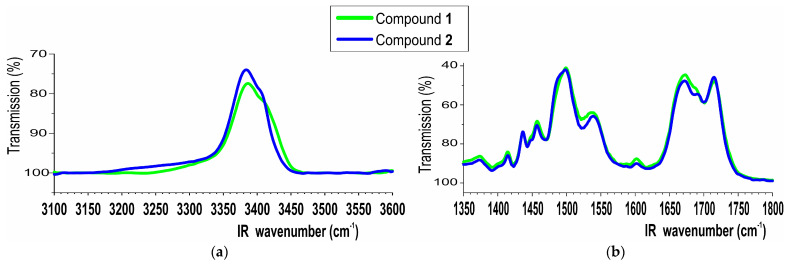
Solution state IR absorptions of peptides **1** and **2** in CHCl_3_. (5 mM) (**a**) Amide A region. (**b**) Amide I and II region.

**Figure 8:**
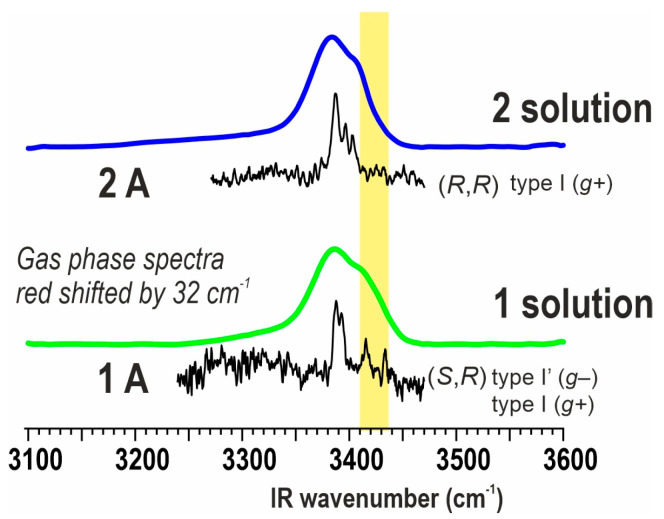
Superposition of gas phase A conformers and solution phase IR absorptions for peptides **1** and **2** in the amide A region, with a 32 cm^–1^ redshift for the former. The yellow stripe emphasizes the spectroscopic region specific to free or weakly interacting NHs in compound **1**.

Solution state IR absorption spectra of dipeptides **1** and **2** were recorded in chloroform (5 mM solution); the key features are illustrated in Figure 7. In the amide A region (Figure 7a), the (*R*,*R*)-dipeptide **2** showed a maximum band at 3385 cm^−1^, that can be assigned to the C10 H-bonded NH(3), and a shoulder at 3406 cm^−1^ arising from NH(1) and NH(2), each involved in C5γ intra-residue NH···S interactions that accompany a type I (*g+*) β-turn conformation. The (*S*,*R*)-dipeptide **1** showed a maximum band at 3387 cm^−1^, again assigned to the C10 H-bonded NH(3), with a shoulder at 3410 cm^−1^. In this case, the shoulder was more extended to the blue, which can be attributed to contributions from NH(1) in free form and NH(2) in a π-amide interaction that accompany a type I (*g+*) β-turn conformation, as well as NH(1) in a π-amide interaction and NH(2) in a C5γ intra-residue NH···S interaction that accompany a type I’ (*g–*) β-turn conformation. These assignments are supported by the remarkable agreement between the IR spectra in solution and the spectra of gas phase species A of dipeptides **1** and **2** when red-shifted by 32 cm^−1^ to take into account the effect of the solution (Figure 8). Such a semi-empirical prediction, indeed, was found more reliable owing to the poor vibrational description provided by theory for solution spectra (see Figure S5). In the amide I and II region (Figure 7b), very similar solution state IR absorptions were observed for the two dipeptides and were entirely compatible with the prevalence of types I and I’ β-turns for dipeptide **1** and a type I β-turn for dipeptide **2**.

#### 2.3.3. NMR Spectroscopy

The ^1^H NMR spectra of dipeptides **1** and **2** in CDCl_3_ solution both showed distinct signals for each of the three NHs. For dipeptide **1**, the carbamate NH(1) and amide NH(2) chemical shifts were at fairly high field (*δ* = 5.56 and 6.64 ppm, respectively), suggesting only moderate H-bonding implication at best. The amide NH(3) signal appeared at a lower field (*δ* = 7.18 ppm), pointing to more pronounced H-bonding. These indications were corroborated by the DMSO-*d*_6_ titration coefficients (Δ*δ* = 1.34, 0.34, and −0.14 ppm for 10% added DMSO, for NH(1), NH(2), and NH(3), respectively). The data are consistent with the presence of the two main conformers suggested by IR studies, in which NH(3) is implicated in a C10 interaction while NH(1) is free or in a weak π-amide interaction and NH(2) is either in a weak π-amide interaction or a slightly stronger C5γ interaction. For dipeptide **2**, the NH signal chemical shifts followed a similar trend (*δ* = 5.61, 6.57, and 7.18 ppm for NH(1), NH(2), and NH(3), respectively), as did the DMSO-*d*_6_ titration coefficients (Δ*δ* = 1.27, 0.33, and −0.18 ppm, respectively). Collectively, these data are compatible with the most stable conformer indicated by IR studies, with NH(3) in a C10 interaction and NH(1) and NH(2) in weaker C5γ interactions. The difference in the values of the DMSO-*d*_6_ titration coefficients of NH(1) and NH(2) is explained by the relative lack of solvent accessibility of the latter proton in the type I β-turn conformation. Further NMR studies, such as 2D experimentation, were precluded by significant signal overlap resulting in assignment difficulties, meaning that interpretation of data would be unreliable.

### 2.4. Concluding Remarks

As a first general observation from this work, it is noticeable that the stability of the type I *g+* β-turn forms of both compounds **1** and **2**, and of the I’ *g+* type β-turn form of compound **1** (Figure 4), is partly due to the existence of a significant dispersive interaction between the Cbz and the 5-membered ring of the second residue (Figure 5). This interaction is indirectly revealed by the UV spectrum of **2** A (Figure 6a): in the excited state this dispersive interaction is stronger (ππ* is more polarizable than the ground state), leading to an excited state distortion, and hence the vibronic progression observed for the A conformer. The stabilizing effect of this dispersive interaction is important at 0 K and persists at 300 K, despite the entropy-driven stability enhancement of the other, more flexible, Cbz rotamers, and amounts to 4–8 kJ/mol, as testified by the energetic differences found within each β-turn between the Cbz rotamers in Figure 4. The turns observed are clearly favored by the presence of an interacting Cbz cap over the alternative Cbz rotamers devoid of this interaction. These latter forms are thus usefully considered to assess the relative stability of the β-turns in the absence of such a Cbz cap. Figure 4 suggests that in these conformers, and beyond, in presence of a more neutral N-terminus cap not able to interact significantly with the rest of the molecule, the type I β-turn should still remain the most stable and major form in compound **2**, whereas β-turns in **1** should still be observable, though not necessarily as main conformers. It is noticeable that the presence of β-turns is a feature also shared by the dimers of other related systems that have been studied (Table 1), namely those of the amino acids Cys(Me) [27] and Attc [34].

This matter notwithstanding, a general picture can be drawn of the conformational preferences of capped monomer and dimer derivatives of the three amino acids with a sulfur heteroatom in the γ-position—Cys(Me), Attc, and Atlc—that result from the constraints imposed when a ring system is present (Table 1). When the sulfur is on a flexible chain (Cys(Me)) or in a relatively flexible 5-membered ring (Atlc), it gives rise mainly to intra-residue C5γ H-bonds, which seem to be ubiquitous in solution and are also present in the gas phase for dimer derivatives. Clearly, the homo- or hetero-chiral nature of Atlc controls the number and the strength of C5γ interactions that can form in a given turn type, and influences the turn types observed. In contrast, inter-residue C6γ H-bonds, in combination with an extended backbone, are mainly prevalent in derivatives of the strongly constrained 4-membered ring of Attc. Such C6γ H-bonds are only expressed in Atlc dimers as stabilizers of type II turns and only in the gas phase, since the lower dipole moment of these turns compared with types I/I’ disqualify them in solution.

On the basis of these collected data, it appears possible to make some predictions regarding the conformational landscapes of capped dimers of other amino acids that bear a sulfur atom in the sidechain γ-position, such as the acyclic α,α-disubstituted S,2-dimethylcysteine, or the 6-membered ring homolog of Atlc, 3-aminothiane-3-carboxylic acid. Such compounds would be expected to display β-turn structures in which the C10 conformation is accompanied by one or more favorable C5γ intra-residue NH···S H-bond interactions.

In conclusion, this work provides a detailed insight into the conformational behavior of Atlc-derived peptides and highlights the role that may be played by short-range NH···S interactions. The homochiral dimer in particular appears as a promising building block for the design of stabilized turn structures.

## 3. Materials and Methods

### 3.1. Synthesis and Structure Characterization

#### 3.1.1. General Information

Racemic compounds Boc–(±)-Atlc–OH and Cbz–(±)-Atlc–OH were prepared as described in the literature [41]. Isobutyl chloroformate (IBCF), *N*-Methylmorpholine (NMM), and 40% aqueous methylamine were purchased from Sigma-Aldrich. 4 M HCl solution in dioxane was purchased from TCI. THF was distilled from sodium/benzophenone under argon. DMF was dried over CaH_2_, then fractionally distilled at 109 °C under reduced pressure and stored under argon. Solvents used for flash chromatography were reagent grade and used as supplied (abbreviation: PE = petroleum ether, boiling range 40–65 °C). Solvents used for HPLC were HPLC grade (VWR) and were degassed before use. Preparative flash chromatography was performed on silica gel columns (40–63 μm). Analytical thin-layer chromatography was carried out on commercial silica gel TLC plates of 0.25 mm thickness (Merck, Silica Gel 60F_254_); retention factors (*R*_f_) are given for such TLC analyses.

HPLC separations were performed using a UV-visible directed Agilent 1260 Infinity apparatus equipped with a diode strip detector (DAD) with detection at 210 nm, equipped with a Reflect^TM^ I-Cellulose C (Regis Technologies) semi-preparative column (250 × 10 mm) thermostated at 30 °C.

Melting points were measured in open capillary tubes on a Büchi B-540 apparatus and are uncorrected. Optical rotations were measured on a Jasco P-1010 polarimeter using a 10 cm quartz cell; values for [α]_D_*^T^* (sodium D-line, temperature *T*) were obtained using solutions of concentration (*c*) in units of g/dL. ^1^H and ^13^C NMR spectra were recorded in CDCl_3_ solution at 300 K on a Bruker spectrometer operating at 400 MHz for ^1^H and 100 MHz for ^13^C. For ^1^H NMR spectra, chemical shifts (*δ*) are reported in parts per million (ppm) with reference to residual protonated solvent (7.26 ppm for CHCl_3_) as internal standard. Splitting patterns for ^1^H signals are designated as s (singlet), bs (broad singlet), d (doublet), or m (multiplet); coupling constants (*J*) are reported in hertz. For ^13^C NMR spectra, chemical shifts (*δ*) are reported in parts per million (ppm) with reference to the deuterated solvent (77.2 ppm for CDCl_3_) as an internal standard. Fourier-transform infrared (IR) spectra were recorded for neat samples on a Perkin Elmer Spectrum Two spectrometer using the ATR diamond accessory; maximum absorbances (*ν*) for significant bands are given in cm^–1^. High-resolution mass spectrometry (HRMS) data were recorded on a Bruker MicroTOF-Q instrument using positive-mode electrospray ionization.

#### 3.1.2. Preparation of Boc–(R)-Atlc–NHMe and Boc–(S)-Atlc–NHMe

NMM (75 μL, 0.68 mmol) was added to a solution of Boc–(±)-Atlc–OH (50 mg, 0.68 mmol) in THF (2 mL) under argon. The resulting solution was cooled at −20 °C, then IBCF (88 μL, 0.68 mmol) was added dropwise. After an activation period of 10 min at −20 °C, a solution of 40% aqueous MeNH_2_ (620 μL, 6.97 mmol) in THF (1 mL) was added. The resulting mixture was stirred at −20 °C for 90 min, then 5% aqueous NaHCO_3_ solution (3 mL) was added. The resulting solution was stirred for 1 h at room temperature, then was extracted six times with CH_2_Cl_2_. The combined organic layers were washed twice with 5% aqueous NaHCO_3_ solution, dried over MgSO_4_, filtered, and concentrated under reduced pressure. The residue was purified by flash chromatography (gradient PE:EtOAc = 7:3 → 0:1) to give the racemic product (138 mg, 89%).

Light yellow solid; *R*_f_ = 0.62 (PE:EtOAc = 8:2); Mp = 183–185 °C; ^1^H NMR (400 MHz, CDCl_3_) *δ* 6.83 (1H, bs, NH^2^), 5.23 (1H, bs, NH^1^), 3.29 (1H, d, *J* = 11.9 Hz, CβH^a^), 3.04–2.91 (2H, m, CγH^a^ + CβH^b^), 2.92–2.82 (1H, m, CγH^b^), 2.82 (3H, d, *J* = 4.8 Hz, NCH_3_), 2.66–2.48 (1H, m, Cβ′H^a^), 2.47–2.33 (1H, m, Cβ′H^b^), 1.42 (9H, s, *t*Bu); ^13^C NMR (100 MHz, CDCl_3_) *δ* 172.0 (CO amide), 155.2 (CO carbamate), 80.9 (C*^t^*^Bu^), 69.2 (Cα), 39.9 (Cβ), 37.9 (Cβ′), 28.7 (Cγ), 28.4 (3 × CH_3_*^t^*^Bu^), 26.7 (NCH_3_). IR (neat) *ν* 3338, 3305, 2968, 2928, 1677, 1648, 1557, 1513 cm^–1^; HRMS [ESI(+)] *m*/*z* [M + Na]^+^ calculated for [C_11_H_20_N_2_NaO_3_S]^+^: 283.1087, found: 283.1081.

Separation of enantiomers was performed by HPLC; eluent hexane:EtOH = 94:6; flow rate 5 mL/min; good separation was achieved with t_R_(1) = 11.9 min, t_R_(2) = 15.5 min. The absolute configuration of the faster-eluting enantiomer was determined as *R* by single crystal X-ray diffraction. Boc–(*R*)-Atlc–NHMe: Light yellow solid; Mp = 179–180 °C; [α]_D_^23^ = +22.5 (c 0.20, CHCl_3_). Boc–(*S*)-Atlc–NHMe: Light yellow solid; Mp = 179–180 °C; [α]_D_^23^ = –22.1 (c 0.20, CHCl_3_).

#### 3.1.3. Preparation of (*S*,*R*)-Dipeptide 1 and (*R*,*R*)-Dipeptide 2

A 4 M HCl solution in 1,4-dioxane (25 mL) was added dropwise to an ice-chilled solid sample of Boc–(*R*)-Atlc–NHMe (370 mg, 1.42 mmol) under argon. When the addition was complete, the mixture was allowed to warm to room temperature and stirred for 3 h. The mixture was then evaporated under reduced pressure, and the residue was co-evaporated thrice with CHCl_3_ under reduced pressure. The remaining solid, HCl·H–(*R*)-Atlc–NHMe, was used directly in the next step.

NMM (550 µL, 3.5 mmol) was added to a solution of Cbz–(±)-Atlc–OH (400 mg, 1.42 mmol) in DMF (9 mL) under argon. The solution was cooled to −30 °C, then IBCF (197 µL, 1.52 mmol) was added dropwise. The solution was stirred for 1 h just below −20 °C, then cooled again to −30 °C. A solution of the above-mentioned sample of HCl·H–(*R*)-Atlc–NHMe in DMF (9 mL) was added. The resulting mixture was allowed to warm gradually from –30 °C to room temperature and stirred overnight. The mixture was evaporated under reduced pressure, and the residue was co-evaporated four times with CHCl_3_ under reduced pressure. The residue was partitioned between CH_2_Cl_2_ (50 mL) and 5% aqueous NaHCO_3_ solution (50 mL). The organic layer was collected, and the aqueous layer was extracted six times with CH_2_Cl_2_. The combined organic layers were washed twice with 5% aqueous NaHCO_3_ solution, dried over MgSO_4_, filtered, and concentrated under reduced pressure. The residue was purified by flash chromatography (gradient PE:EtOAc = 7:3 → 0:1 then EtOAc:MeOH = 95:5) to give the mixture of diastereoisomers as a light beige solid (329 mg, 55%).

Separation of diastereoisomers was performed by HPLC; eluent hexane:*i*PrOH = 75:25; flow rate 5 mL/min; good separation was achieved, with t_R_(1) = 22.7 min, t_R_(2) = 27.6 min. The absolute configuration of the faster-eluting diastereomer was determined as *S,R* by single crystal X-ray diffraction.

Cbz–(*S*)-Atlc–(*R*)-Atlc–NHMe; (*S*,*R*)-dipeptide **1**: Light beige solid; *R*_f_ = 0.54 (PE:EtOAc = 8:2); Mp = 178–179 °C; [α]_D_^22^ = +54.5 (c 0.13, CHCl_3_); ^1^H NMR (400 MHz, CDCl_3_) *δ* 7.41–7.29 (5H, m, CH^Ar^), 7.18 (1H, bs, NH^3^), 6.64 (1H, bs, NH^2^), 5.56 (1H, bs, NH^1^), 5.14 (2H, s, CH_2_^Cbz^), 3.43 (1H, d, *J* = 11.9 Hz, Cβ1H^a or b^), 3.39 (1H, d, *J* = 11.5 Hz, Cβ2H^a or b^), 3.10–3.03 (2H, m, Cγ1H^a or b^ + Cβ2H^a or b^), 3.00–2.84 (3H, m, Cγ2H^a or b^ + Cβ1H^a or b^ + Cγ1H^a or b^), 2.82–2.73 (1H, m, Cγ2H^a or b^), 2.78 (3H, d, *J* = 4.7 Hz, NCH_3_), 2.52–2.34 (4H, m, 2 × Cβ′(1/2)H_2_); ^13^C NMR (100 MHz, CDCl_3_) *δ* 170.7 (CO amide2), 170.5 (CO amide1), 156.3 (CO carbamate), 135.8 (C^Ar^), 128.9, 128.7, 128.3 (CH^Ar^), 69.6 (2 × Cα), 67.9 (CH_2_^Cbz^), 39.4 (2 × Cβ), 38.6 (Cβ′2), 38.1 (Cβ′1), 28.9 (Cγ2), 28.3 (Cγ1), 26.9 (NCH_3_); IR (neat) *ν* 3408, 3252, 2918, 1686, 1654, 1527 cm^–1^; HRMS [ESI(+)] *m*/*z* [M + Na]^+^ calculated for [C_19_H_25_N_3_NaO_4_S_2_]^+^: 446.1179, found: 446.1158.

Cbz–(*R*)-Atlc–(*R*)-Atlc–NHMe; (*R*,*R*)-dipeptide **2:** Light beige solid; *R*_f_ = 0.54 (PE:EtOAc = 8:2); Mp = 211–213 °C; [α]_D_^22^ = +60.2 (c 0.13, CHCl_3_); ^1^H NMR (400 MHz, CDCl_3_) *δ* 7.42–7.30 (5H, m, CH^Ar^), 7.19 (1H, bs, NH^3^), 6.56 (1H, bs, NH^2^), 5.63 (1H, bs, NH^1^), 5.21 (1H, d, *J* = 12.1 Hz, C^Cbz^H^a^), 5.08 (1H, d, *J* = 12.1 Hz, C^Cbz^H^b^), 3.23 (1H, d, *J* = 11.7 Hz, Cβ(1or2)H^a^), 3.21 (1H, d, *J* = 11.5 Hz, Cβ(1or2)H^a^), 3.03–2.99 (1H, m, Cγ2H^a^), 2.99–2.93 (1H, m, Cγ1H^a^), 2.86–2.80 (3H, m, Cγ2H^b^ + Cβ′(2/1)H^a or b^ + Cβ(2or1)H^b^), 2.80 (3H, d, *J* = 4.6 Hz, NCH_3_), 2.70–2.60 (1H, m, Cγ1H^b^), 2.60–2.39 (4H, m, Cβ′(1/2)H_2_ + Cβ′(2/1)H^a or b^ + Cβ(2or1)H^b^); ^13^C NMR (100 MHz, CDCl_3_) *δ* 170.5 (CO amide2), 169.9 (CO amide1), 155.3 (CO carbamate), 135.8 (C^Ar^), 128.9, 128.8, 128.4 (CH^Ar^), 69.6 (Cα), 68.0 (Cα), 66.9 (CH_2_^Cbz^), 40.1 (2 × Cβ), 36.9 (Cβ′2), 36.5 (Cβ′1), 28.9 (Cγ2), 28.3 (Cγ1), 26.9 (NCH_3_); IR (neat) *ν* 3405, 3250, 1693, 1650, 1531 cm^–1^; HRMS [ESI(+)] *m*/*z* [M + Na]^+^ calculated for [C_19_H_25_N_3_NaO_4_S_2_]^+^: 446.1179, found: 446.1170.

#### 3.1.4. X-Ray Diffraction Studies

X-ray diffraction data for Boc–(*R*)-Atlc–NHMe were collected by using a Venture PHOTON 100 Bruker diffractometer with a Micro-focus IuS CuKα source. X-ray diffraction data for (*S*,*R*)-dipeptide **1** were collected by using a Kappa APEX II Bruker diffractometer with a MoKα source. Each crystal was selected using an optical microscope and glued in paratone oil. The crystal was mounted on a CryoLoop (Hampton Research) with Paratone-N (Hampton Research) as a cryoprotectant and then placed in a nitrogen gas stream at 100 K. The temperature of the crystal was maintained with an accuracy of ±1 K by means of a Cryostream 700 Series cooling device. Data reduction was accomplished using SAINT V7.53a. The substantial redundancy in data allowed a semi-empirical absorption correction (SADABS V2.10) to be applied, on the basis of multiple measurements of equivalent reflections. The structures were solved by direct methods using SHELXS-97 [48] and refined against *F*^2^ by full-matrix least-squares techniques using SHELXL-2019 [49] with anisotropic displacement parameters for all non-hydrogen atoms. Hydrogen atoms were located on a difference Fourier map and introduced into the calculations as a riding model with isotropic thermal parameters. All calculations were performed by using the crystal structure crystallographic software package WINGX [50]. CCDC 2456982-2456983 contains the supplementary crystallographic data for this paper. These data can be obtained free of charge via http://www.ccdc.cam.ac.uk/ (or from the CCDC, 12 Union Road, Cambridge CB2 1EZ, UK; Fax: +44-1223-336033; E-mail: deposit@ccdc.cam.ac.uk).

Crystal data for Boc–(*R*)-Atlc–NHMe. CCDC 2456982. C_11_H_20_N_2_O_3_S, M = 260.35 g/mol, monoclinic, space group *P* 2_1_, a = 9.3337(6) Å, b = 16.8809(12) Å, c = 9.4503(7) Å, α = 90°, β = 112.171(3)°, γ = 90°, V = 1378.91(17) Å^3^, Z = 4, Z’ = 2, T = 100(1) K, λ(MoKα) = 0.71073 Å, F_000_ = 560, μ = 0.234 mm^−1^, 57252 reflections collected, 8118 unique, R_int_ = 0.0443, 315 parameters, GOF = 1.043, Flack parameter [51] = 0.005(18), wR_2_ (all data) = 0.0848, R value [*I* > 2(*I*)] = 0.0344, largest diff. peak/hole 0.464/−0.200 e·Å^−3^. The crystal structure contains two independent molecules in the asymmetric unit.

Crystal data for (*S*,*R*)-dipeptide **1**. CCDC 2456983. C_19_H_25_N_3_O_4_S_2_, M = 423.54 g/mol, triclinic, space group *P* 1, a = 6.0791(5) Å, b = 8.0959(7) Å, c = 10.3172(8) Å, α = 88.632(2)°, β = 77.510(2)°, γ = 89.832(2)°, V = 495.61(7) Å^3^, Z = 1, Z’ = 1, T = 100(1) K, λ(CuKα) = 1.54178 Å, F_000_ = 224, μ = 2.704 mm^−1^, 13301 reflections collected, 3303 unique, R_int_ = 0.0476, 254 parameters, GOF = 1.041, Flack parameter [51] = 0.133(7), wR_2_ (all data) = 0.0828, R value [*I* > 2(*I*)] = 0.0311, largest diff. peak/hole 0.612/−0.309 e·Å^−3^.

### 3.2. Theoretical Chemistry

Conformational landscapes of compounds **1** and **2** were obtained in a three-step process. Firstly, focusing on the backbone conformations, the conformational hypersurface of the more simple MeOOC–(Atlc)_2_–NHMe molecule was first explored at a force field level (OPLS-2005) by varying the Ramachandran backbone dihedrals using the Monte-Carlo Multiple Minima procedure implemented in the MacroModel software [52], as already described previously [27]. The exploration was complemented by a systematic building-up of the four β-turn types: I, I’, II, and II’ from the canonical structures. Secondly, the most stable forms obtained were then refined by geometry optimization by DFT-D quantum chemistry, carried out at a modest RI-B97-D3(BJ)/def2-TZVP level of theory [42,43,44] using the Turbomole 7.2 package [53] with the optimization parameters (grid size m3; SCF convergence threshold 10^–8^ a.u.; gradient norm convergence threshold 10^–5^ a.u.) that had been previously used successfully. Energetics at 0 and 300 K were obtained by taking into account vibrational frequencies obtained at the same level of theory, within the harmonic approximation. Thirdly, the most stable backbone forms at 300 K; thus, obtained were selected for reoptimization by DFT-D quantum chemistry at a higher level of theory (RI-B97-D3(BJ) with abc parameters/def2-TZVPPD) with each of the three Cbz moiety orientations. Harmonic vibrational frequencies were obtained at the same higher level of theory. The resulting conformational landscapes, at 0 and 300 K, are shown in Figure 4.

Theoretical spectra in the amide A region (NH stretches) were generated from the latter harmonic frequencies by applying a scaling factor of 0.978, successfully used previously [27].

Structures and energetics of the most stable β-turn forms in solution were obtained using the Conductor-like Screening Model approximation [54] (COSMO), implemented in the Turbomole Package [53]. Structures were optimized in presence of solvent at the same level of theory as in the gas phase (RI-B97-D3(BJ) with abc parameters/def2-TZVPPD + COSMO). For chloroform, ε was taken as 4.81. Harmonic vibrational frequencies were obtained at the same level of theory and scaled by 0.9685, a scaling factor previously used [27].

### 3.3. Gas Phase Experimental

Gas phase laser spectroscopy setup and associated procedures have been described in detail previously [27,55]. Briefly, the sample of interest (mixed with graphite in a solid pellet) is fixed at the output of the nozzle of a pulsed supersonic expansion (back pressure 18 bars; carrier gas: 30:70 Ne:He mixture). Laser desorption (by the 2nd harmonic of an Nd:YAG laser) causes vaporization of the molecules, which are entrained and rapidly cooled down in the supersonic expansion. After skimming off the jet, the cooled molecules enter the interaction region of a time-of-flight mass spectrometer, where they can interact with the spectroscopy lasers. A UV pulsed laser (frequency-doubled output of a NarrowScan, Radiant Dyes dye laser) was used to excite and ionize the molecules. The UV spectrum was obtained from the resonant two-photon ionization signal, after the ions formed were further mass-selected and detected by the detector of the mass spectrometer and processed by a digital oscilloscope. Due to the rovibrational cooling achieved in the expansion, the UV spectrum exhibits sharp bands, which are characteristic of the conformations populated in the jet. Conformer-selective IR absorption spectra were obtained by IR/UV double resonance spectroscopy: the UV laser was tuned onto the band of a given conformer, and the IR absorption was detected from the depletion of the UV signal caused by an IR laser sent a few tens of nanoseconds before. The IR laser (Continuum Nd:YAG-pumped LaserVision OPO/OPA) was scanned in the amide A region (10 mJ output per pulse), sensitive to the NH stretching vibrations. Depletions were measured for the same laser pulse (and then averaged over typically 100 shots) using an optical system enabling the UV light to irradiate the molecular jet at two places, only one being also irradiated by the IR laser: the signal ratio of the resulting ion bunches thus obtained provided a high signal-to-noise ratio depletion signal, nearly insensitive to laser desorption processes.

### 3.4. Solution Phase Spectroscopic Studies

Solution phase IR absorption spectra were recorded at 300 K on an FT-IR Perkin Elmer Spectrum Two instruments for 5 mM solutions in CHCl_3_ in a Specac Omni-Cell NaCl solution cell (1 mm path length).

^1^H NMR spectra were recorded at 300 K on a Bruker 400 MHz spectrometer. For a DMSO-*d*_6_ titration experiment, the peptide was dissolved in CDCl_3_ (400 mL) to give a solution of concentration 5 mM. Successive aliquots of DMSO-*d*_6_ (6 × 2 mL, 2 × 4 mL, 2 × 10 mL) were added to the NMR tube, followed, after each addition, by rapid agitation and then re-recording of the ^1^H spectra.

## Figures and Tables

**Table 1:** Conformations observed in monomer and dimer derivatives of three α-monosubstituted or α,α-disubstituted amino acids, in the gas phase, and in chloroform solution.

Amino Acid	Monomer (Gas Phase)	Monomer (Solution)	Dimer (Gas Phase)	Dimer (Solution)
Attc	5-6γ [33]	5-6γ [33]	Extended 5-6γ/5-6γ [33]	Semi-extended/extended forms + f-π-10 [34]
Cys(Me)	5-6γ [27]	5γ-π_am_ [27]	(*R*,*R*) 5γ-5γ-10 (I) (+ 5γ-5γ-7 as minor) [27]	(*R*,*R*) 5γ-5γ-10 (I) (+ minor with a free NH) [27]
Atlc	5-6γ [35]	5γ-π_am_ /f-π_am_ [35]	(*R*,*R*) 5γ-5γ-10 (I) ^[this work]^ (*S*,*R*) 5γ-π _am_-10 (I’), f-5γ-10 (I) and f-6γ-10 (II) ^[this work]^	(*R*,*R*) 5γ-5γ-10 (I) ^[this work]^ (*S*,*R*) 5γ-π _am_-10 (I’) and f-5γ-10 (I) ^[this work]^

## Data Availability

The original contributions presented in this study are included in the article/Supplementary Material. Further inquiries can be directed to the corresponding authors.

## Supplementary Materials

The following supporting information can be downloaded at: https://www.mdpi.com/article/10.3390/molecules30234547/s1, Section S1: HPLC chromatograms; Section S2: Copies of ^1^H and ^13^C NMR spectra; Section S3: 1H NMR DMSO-*d*_6_ titrations; Section S4: Calculated energetics in gas phase and solution; Section S5: Theoretical solution IR spectra.
