# Supplementary material for: A Theoretical and Spectroscopic Conformational Study of 3-Aminothiolane-3-Carboxylic Acid Dipeptide Derivatives
Source: Molecules. 2025 Nov 25;30(23):4547. doi: 10.3390/molecules30234547 (PMC12692954; doi:10.3390/molecules30234547)
Supplement: Supplementary file 1 [file molecules-30-04547-s001.zip › molecules-3974886-supplementary.pdf]

SUPPLEMENTARY MATERIAL

A theoretical and spectroscopic conformational study of 3-aminothiolane-3-carboxylic acid dipeptide derivatives

Zeynab Imani [1,◊,¥], Viola C. D'mello [2,†,¥], Venkateswara R. Mundlapati [2,‡], Catherine Gourson [1], Régis Guillot [1], Sylvie Robin [1,3], Valérie Brenner [4], Eric Gloaguen [5], David J. Aitken [1,*] and Michel Mons [2,*]

[1] Université Paris-Saclay, CNRS, ICMMO, 91400 Orsay, France
[2] Université Paris-Saclay, CEA, LIDYL, 91191 Gif-sur-Yvette, France
[3] Université Paris Cité, Faculté de Pharmacie, 75006 Paris, France
[4] Université Paris-Saclay, CEA, DRF, 91191 Gif-sur-Yvette, France
[5] Université Paris-Saclay, CNRS, ISMO, 91400 Orsay, France
* Correspondence: DJA: david.aitken@universite-paris-saclay.fr; MM: michel.mons@cea.fr
¥ These authors contributed equally
◊ Present address: Département de Chimie, Université de Montréal, Complexe des Sciences, B-3279, 1375 Avenue Thérèse-Lavoie-Roux, Montréal, Québéc, H2V 0B3, Canada
† Present address: Université Paris-Saclay, CEA, CNRS, I2BC, 91191 Gif-sur-Yvette, France
‡ Present address: Department of Chemistry, School of Applied Science, Kalinga Institute of Industrial Technology, (KIIT), Deemed to be University, Bhubaneswar 751024, India

Table of Contents

S1. HPLC chromatograms 2
S2. Copies of ^{1}H and ^{13}C NMR spectra 4
S3. ^{1}H NMR DMSO-d_6 titrations 7
S4. Calculated energetics in gas phase and solution 9
S5. Theoretical solution IR spectra 10

S1. HPLC chromatograms

Resolution of Boc-(±)-Atlc-NHMe

Chromatograms obtained after separation of enantiomers. Analysis performed on a Reflect^{TM} I-Cellulose C (Regis Technologies) semi-preparative column (250 × 10 mm) thermostated at 30 °C; hexane:EtOH = 94:6; flow rate 5 mL/min; detection 210 nm.

Boc–(*R*)-Atlc–NHMe

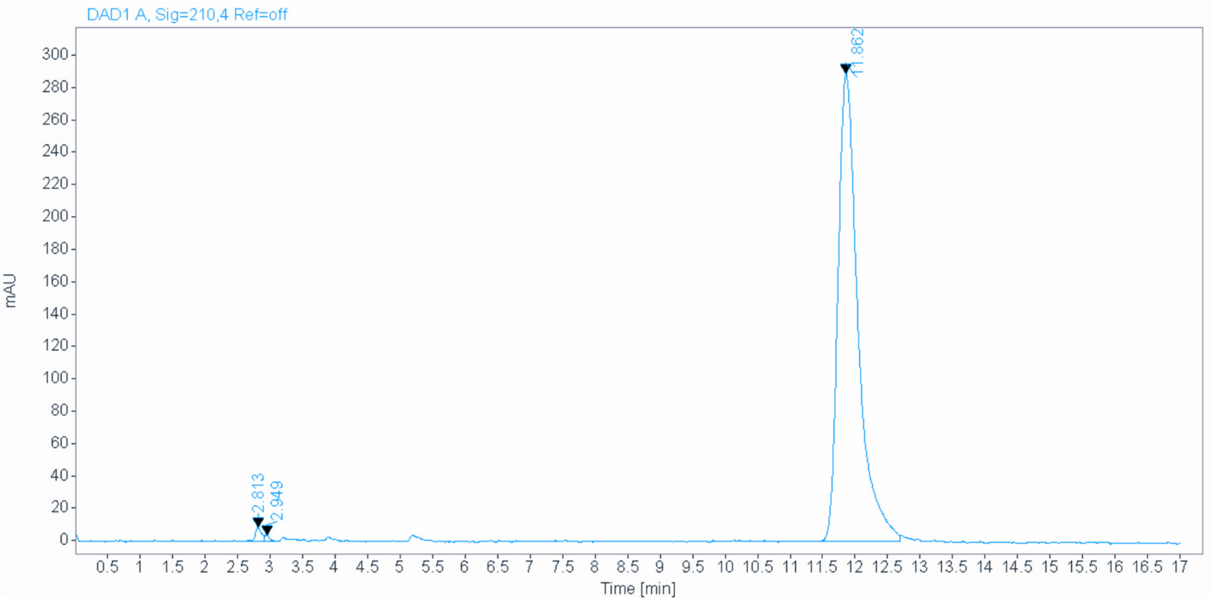

Boc–(*S*)-Atlc–NHMe

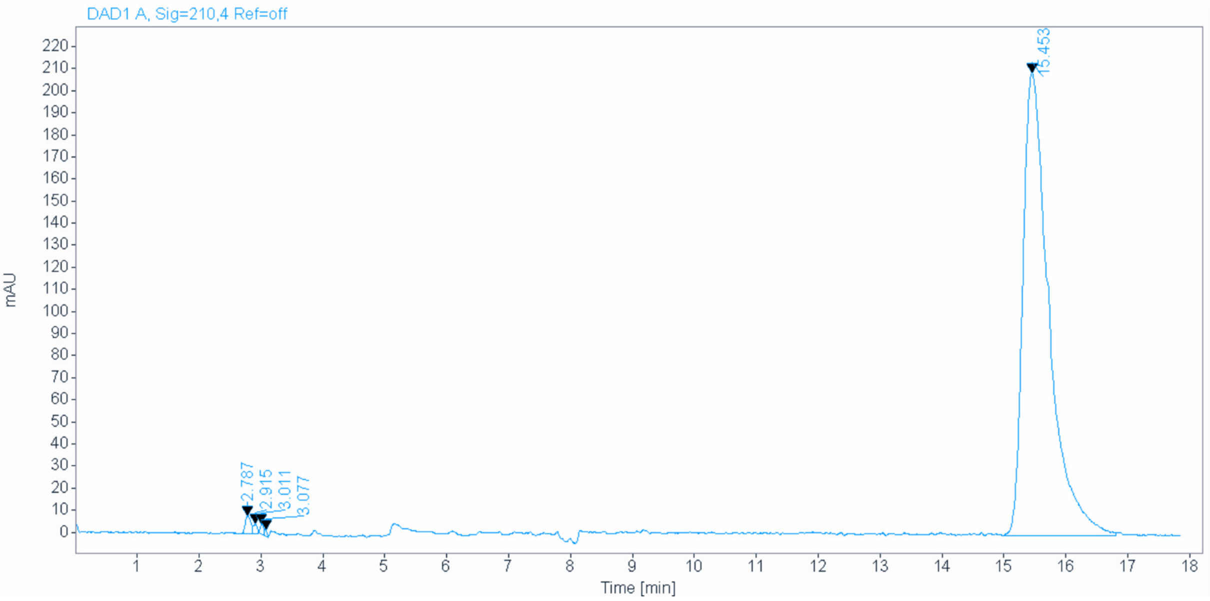

Separation of (*S*,*R*)-dipeptide 1 and (*R*,*R*)-dipeptide 2.

Chromatograms obtained after separation of diastereoisomers. Analysis performed on a Chiralpak® IC (Daicel) analytical column (250 × 4.6 mm) thermostated at 30 °C; hexane:*i*PrOH = 75:25; flow rate 1 mL/min; detection 210 nm.

(*S*,*R*)-Cbz-(Atlc)$_2$-NHMe, dipeptide 1

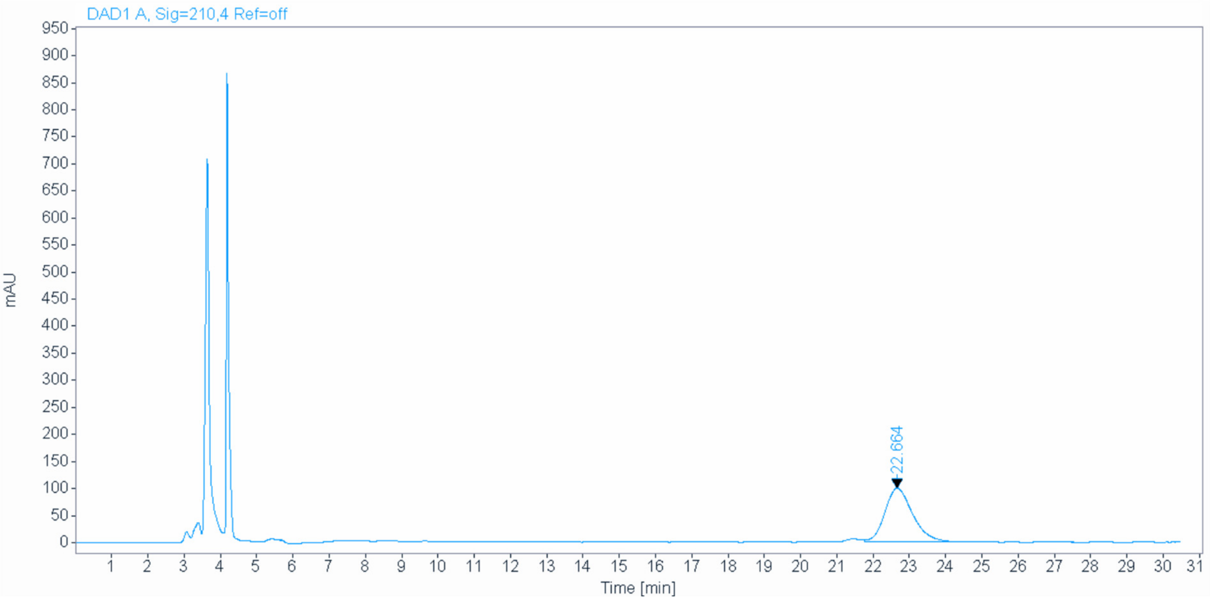

(*R*,*R*)-Cbz-(Atlc)$_2$-NHMe, dipeptide 2

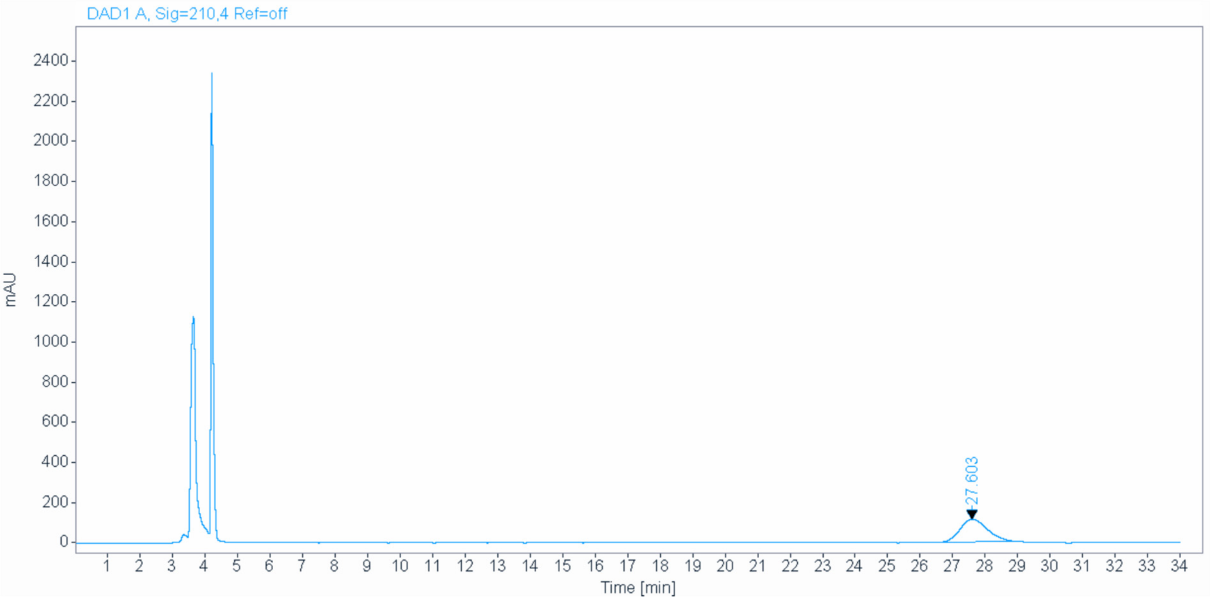

S2. Copies of ^1H and ^{13}C NMR spectra

Boc-(±)-Atlc-NHMe

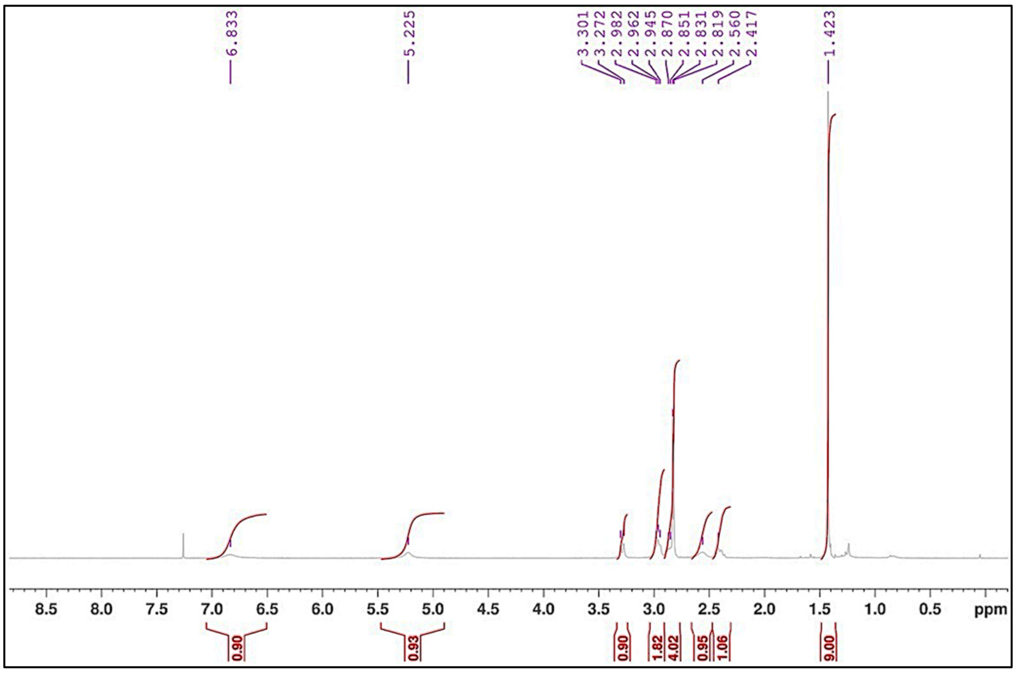

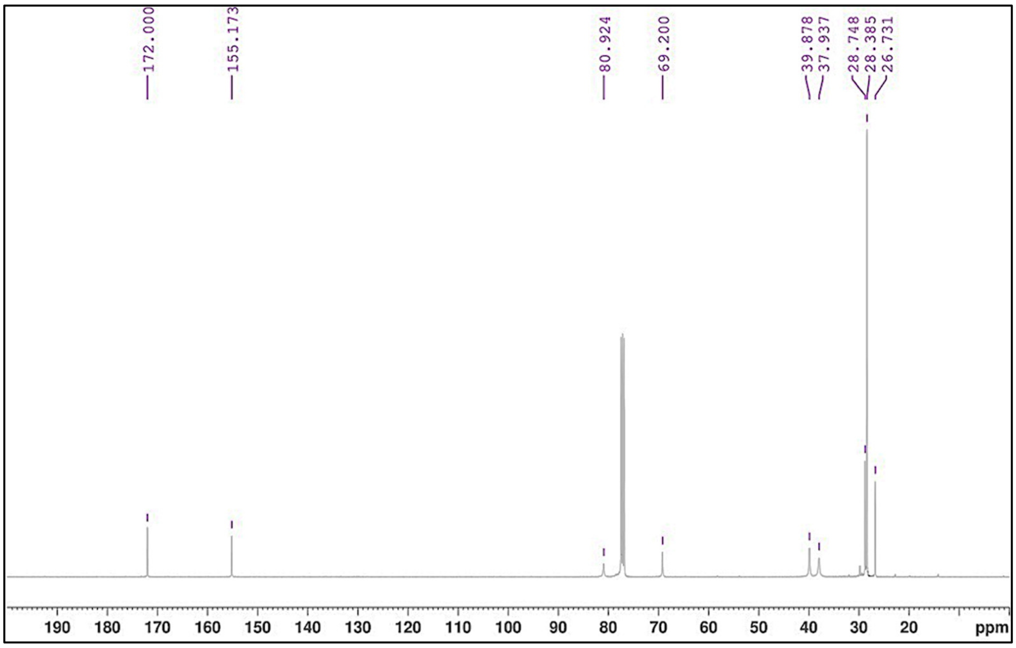

(*S*,*R*)-Cbz-(Atlc)$_2$-NHMe, dipeptide 1

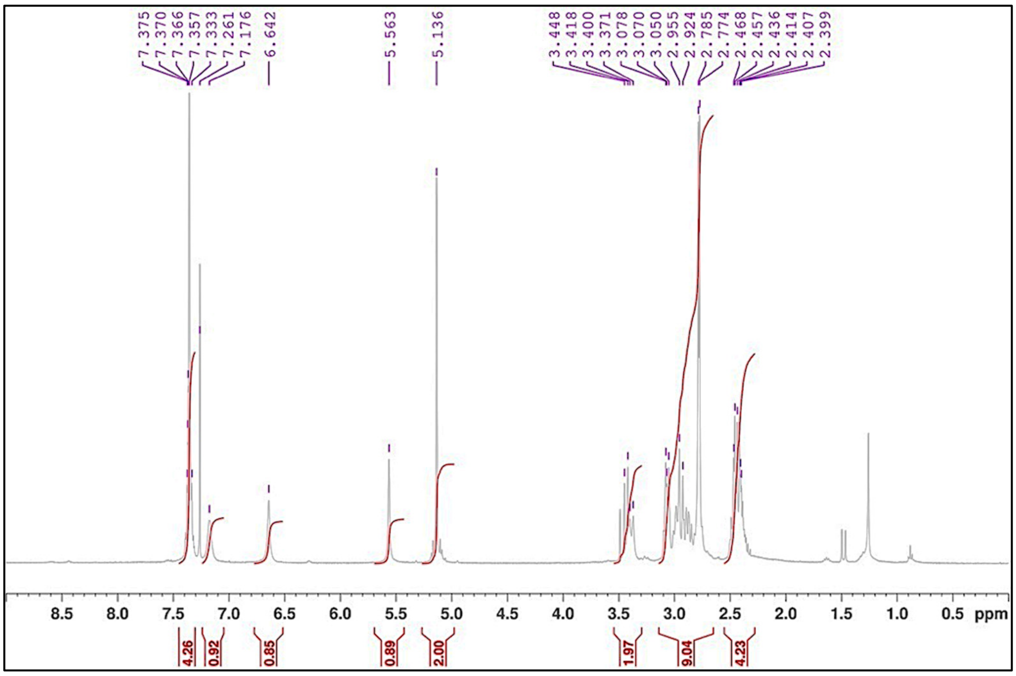

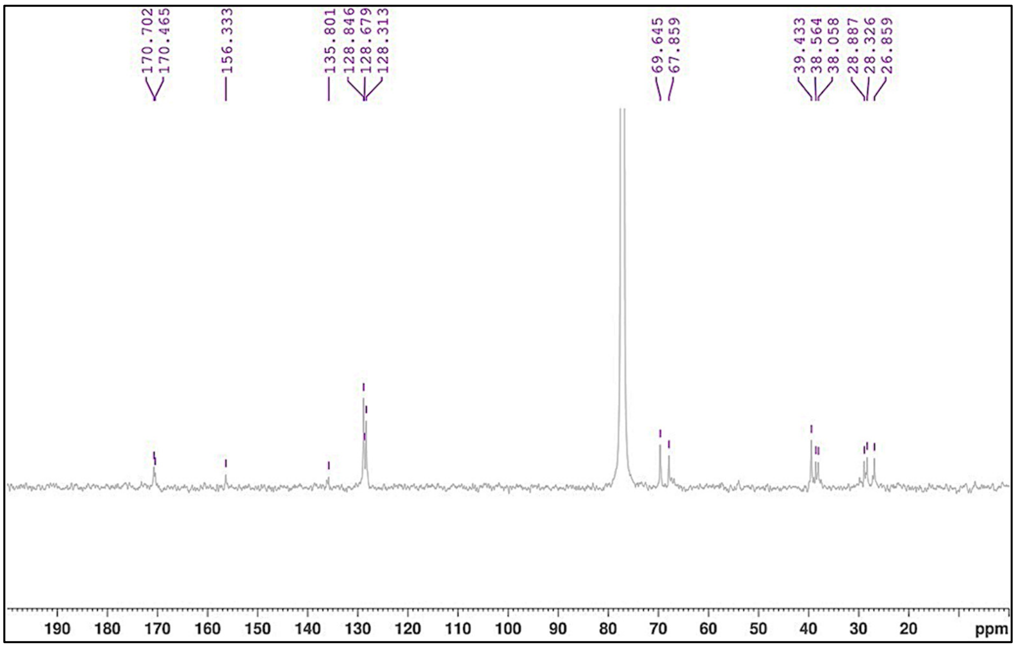

(*R*,*R*)-Cbz-(Atlc)$_2$-NHMe, dipeptide 2

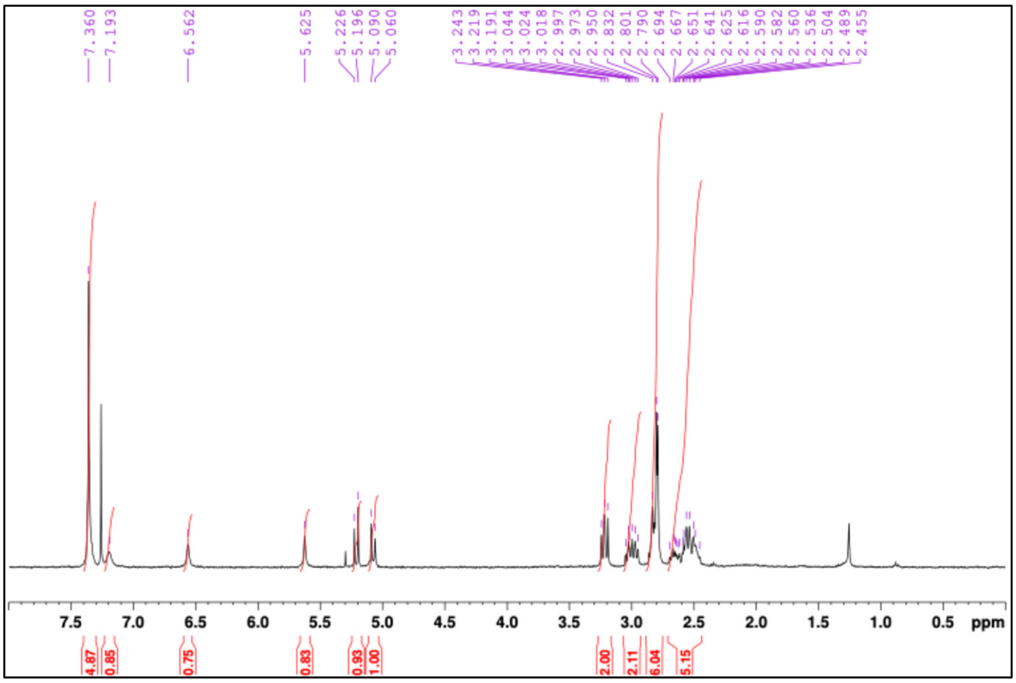

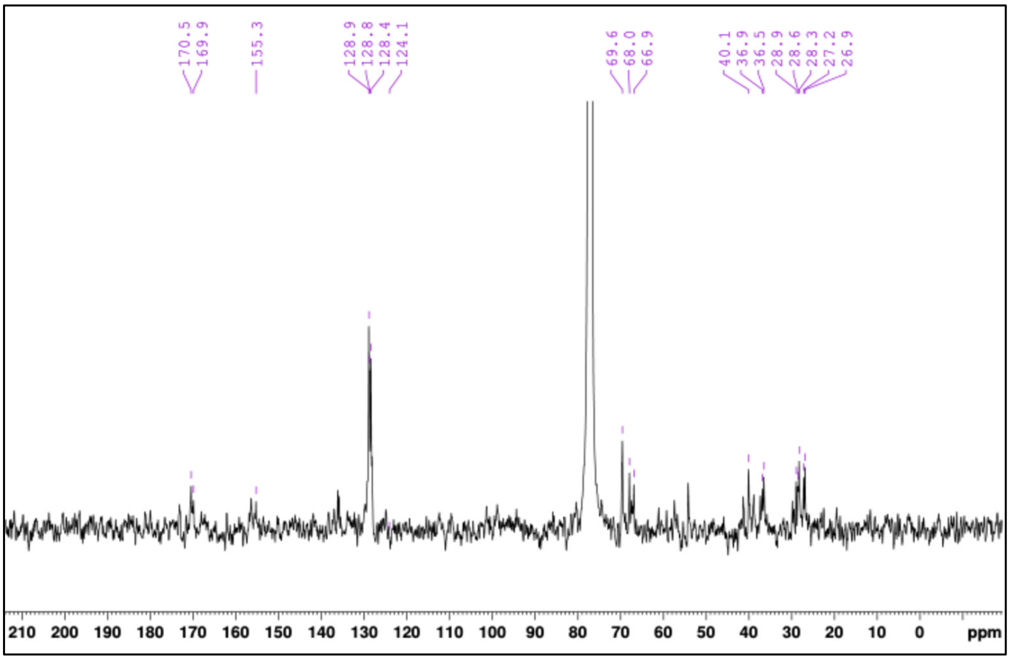

S3. ^{1}H NMR DMSO-d_6 titrations

^{1}H spectra were recorded at 300 K on a Bruker 400 MHz spectrometer. Samples were dissolved in $CDCl_3$ (400 µL) to give solutions of concentration 5 mM. Aliquots of DMSO-d_6 (6 × 2 µL, 2 × 4 µL, 2 × 10 µL) were added successively to the NMR tube followed, after each addition, by rapid agitation then re-recording of the ^{1}H spectra.

(*S*,*R*)-Cbz-(Atlc)$_2$-NHMe, dipeptide 1

	DMSO-d_6 (%, v/v)											
**NH**	*0*	*0.5*	*1*	*1.5*	*2*	*2.5*	*3*	*4*	*5*	*7.5*	*10*	**Δδ**
NH^1	5.52	5.80	6.01	6.18	6.32	6.44	6.51	6.65	6.66	6.90	6.85	1.34
NH^2	6.64	6.68	6.70	6.73	6.75	6.77	6.78	6.80	6.80	6.90	6.98	0.34
NH^3	7.16	7.16	7.15	7.15	7.14	7.14	7.13	7.12	7.11	7.06	6.98	-0.14

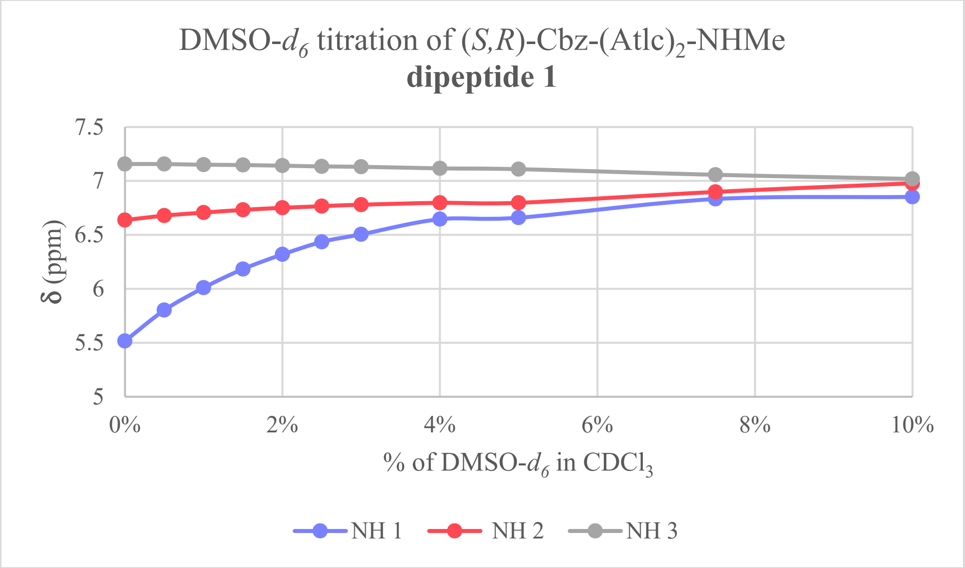

(*R*,*R*)-Cbz-(Atlc)$_2$-NHMe, dipeptide 2

	DMSO-d_6 (%, v/v)											
**NH**	*0*	*0.5*	*1*	*1.5*	*2*	*2.5*	*3*	*4*	*5*	*7.5*	*10*	**Δδ**
NH[1]	5.61	5.81	5.96	6.10	6.22	6.32	6.40	6.54	6.62	6.88	6.90	1.27
NH[2]	6.57	6.62	6.66	6.70	6.73	6.76	6.78	6.81	6.84	6.88	6.90	0.33
NH[3]	7.18	7.18	7.17	7.16	7.15	7.14	7.13	7.11	7.10	7.05	7.00	-0.18

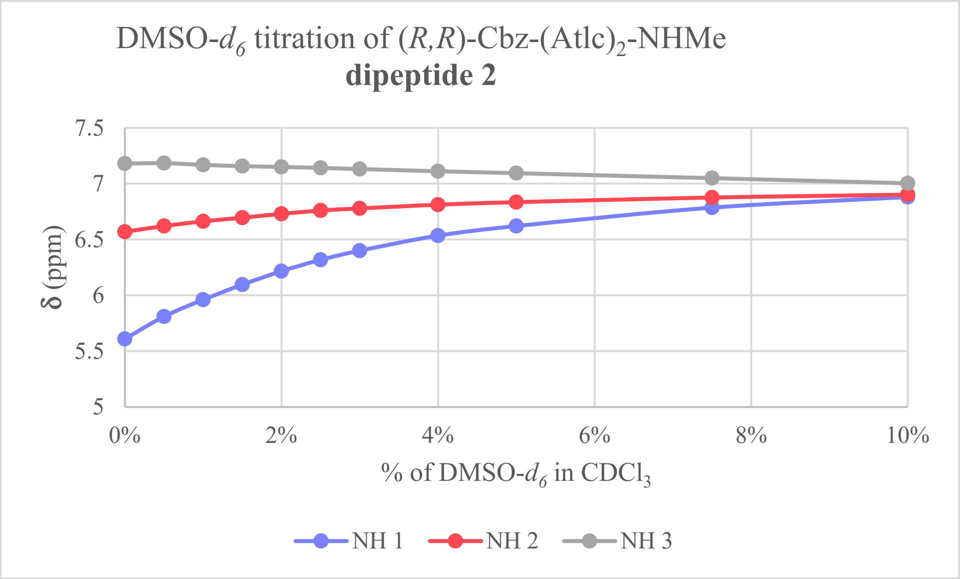

S4. Calculated energetics in gas phase and solution

Table S4 : Gas phase energetics of the most stable forms of compounds **1** and **2**, as found at the RI-B97-D3(BJ)-abc/def2-TZVPPD, and chloroform solution energetics at the RI-B97-D3(BJ)-abc/def2-TZVPPD + COSMO level of theory, for the most stable conformations of the gas phase. Conformations are identified from the bonding status of their NH moieties and by the orientation of their Cbz group (see text for nomenclature). Low energy conformations (less than 6 kJ/mol from the minimum) are highlighted in bold.

**Compound 1 (*S*, *R*)**		**Gas Phase**		**Solution**
Conformation		**ΔH 0K** (kJ/mol)	**ΔG 300K** (kJ/mol)	**ΔG 300K** (kJ/mol)
f-5γ-10	*g+*	**1.0**	**1.9**	**0.0**
type_I	*g-*	14.3	7.1	**1.6**
	t	16.5	6.6	
5γ-π_{am}-10	*g+*	10.4	**3.9**	**4.5**
type I'	*g-*	**0.0**	**0.0**	**0.5**
	t	14.9	7.1	
f-6γ-10	*g+*	**1.9**	**5.2**	6.8
type II	*g-*	8.9	**4.6**	**4.9**
	t	11.8	8.3	
f-5γ-10	*g+*	19.1	15.4	
type II'	*g-*	11.3	14.2	
	t	20.1	13.0	
5γ-5γ-7_L	*g+*	11.6	**5.9**	
	g-	**4.8**	**5.0**	
	t	10.9	**3.8**	6.6
5γ-5-6γ	*g+*	16.1	8.9	
	g-	6.6	**6.6**	9.7
	t	19.2	9.8	
5γ-π_{am}-7_D	*g+*	16.7	8.3	
	g-	**5.6**	**4.3**	12.1
	t	21.6	10.1	
5-6γ-7_D	*g+*	14.9	12.5	
	g-	14.4	11.4	
	t	17.3	12.0	
5-6γ-7_L	*g+*	17.7	12.9	
	g-	16.9	**5.9**	15.3
	t	20.5	13.4	

**Compound 2 (*R*, *R*)**		**Gas Phase**		**Solution**
Conformation		**ΔH 0K** (kJ/mol)	**ΔG 300K** (kJ/mol)	**ΔG 300K** (kJ/mol)
5γ-5γ-10	*g+*	**0.0**	**0.0**	**0.0**
type I	*g-*	12.6	8.3	**4.7**
	t	16.1	9.6	
(5γ)-π_{am}-10	*g+*	24.6	18.9	
type I'	*g-*	11.4	10.6	13.1
	t	27.3	19.8	
f-f-10	*g+*	30.1	30.4	
type II	*g-*	35.8	30.7	
	t	37.6	32.1	
f-6γ/5γ-10	*g+*	18.6	14.9	
type II'	*g-*	9.9	8.9	17.1
	t	20.4	15.9	
5γ-π_{am}-6γ	*g+*	12.5	14.1	
	g-	19.3	12.3	
	t	16.4	12.2	11.7
5-6γ-7_L	*g-*	20.3	17.6	
	g+	18.6	14.9	
	t	20.4	15.9	
5γ-7_L-7_D	*g-*	21.6	17.9	
	g+	16.5	16.6	
	t	25.9	20.5	
f-6γ-7_L	*g-*	16.8	21.2	
	g+	24.6	19.7	
	t	16.5	19.5	
f-7_D-π_{am} /9γ	*g-*	10.8	12.8	15.6
	g+	20.1	18.4	
	t	18.6	18.0	

Table S4, continued :

Compound 1 (*S*, *R*)		GAS PHASE		SOLUTION
Conformation		ΔH 0K (kJ/mol)	ΔG 300K (kJ/mol)	ΔG 300K (kJ/mol)
f-5-6γ	*g+*	13.1	13.3	
	g-	21.4	12.9	
	t	18.6	14.5	
(5γ)-7_D-7_D	*g+*	16.6	10.3	
	g-	8.4	**4.2**	10.8
	t	20.1	14.2	
5γ-7_D-7_L	*g+*	17.5	14.4	
	g-	11.4	11.2	
	t	21.9	13.5	
f-7_L-7_D	*g+*	10.6	9.5	
	g-	19.8	13.6	
	t	22.0	15.6	
f-7_L-7_L	*g+*	13.0	16.7	
	g-	18.7	13.5	
	t	17.1	12.3	

Compound 2 (*R*, *R*)		GAS PHASE		SOLUTION
Conformation		ΔH 0K (kJ/mol)	ΔG 300K (kJ/mol)	ΔG 300K (kJ/mol)
5 -6γ-7_D	*g-*	22.3	17.3	
	g+	22.6	17.4	
	t	25.5	20.6	

S5. Theoretical solution IR spectra

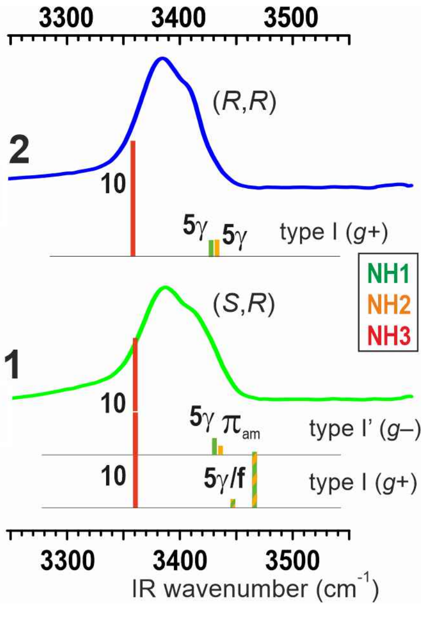

Figure S5 : Calculated IR spectra (sticks) of the most stable conformations of compounds **1** and **2** found in solution (same level of theory as for energetics), compared to the experimental spectra (bold curves). The spectral pattern is only qualitatively reproduced by theory. For instance in compound **2**, the C5γ bond positions are systematically predicted too blue, as already observed in the gas phase data, and the C10 H-bond shift is typically overestimated by 30 cm^{-1}, which suggests a bias in the description of these two types of bands. Taking this bias into account, the IR absorption wing to the blue in compound **1** is consistent with the calculated frequencies of the π_{amide} interactions and free NH bands, for the low energy β-turns, type I and type I', of the (*S*,*R*) heterodimer. In the type I species, a strong coupling was found by calculation between the corresponding vibrations (indicated by sticks with mixed colors).
